# Editorial: Advancing *in vitro* cell culture practices: achieving truly animal-free experiments and scientifically reliable and reproducible methods

**DOI:** 10.3389/ftox.2026.1892833

**Published:** 2026-09-21

**Authors:** Atena Malakpour-Permlid, Stina Oredsson, Tilo Weber

**Affiliations:** 1 Department of Biochemistry and Molecular Biology, University of Southern Denmark, Odense, Denmark; 2 Department of Biology, Lund University, Lund, Sweden; 3 Animal Welfare Academy of the German Animal Welfare Federation, Neubiberg, Germany

**Keywords:** animal ethics, animal-free antibodies, animal-free materials, basement membrane extract (matrigel), fetal bovine serum (FBS), reproducibility, serum-free, xeno-free cell culture

## Introduction

Culturing cells outside the body is integral to a wide range of scientific fields and has undisputedly contributed to the advancement of cell biology and physiological knowledge. However, despite these positive achievements, cell culture protocols still rely on animal-derived components, primarily fetal bovine serum (FBS), but also basement membrane extracts, collagen, gelatin, trypsin, antibodies, and many other materials of animal origin (van der Valk et al., 2010; Duarte et al., 2023; Modi, 2025; Reichstein et al., 2026). The challenge is not the protocols *per se*, but the use of animal-derived products, which are associated with several well-recognized ethical concerns and scientific limitations. The ethical concerns surrounding animal-derived materials are evident, since obtaining them often causes animals to suffer or to be killed. For example, FBS is produced from blood obtained from the fetuses of slaughtered cows. However, pregnant cows should not be transported to abattoirs in the first place and thus neither dam nor fetus would be killed. This would prevent fetal blood from occurring as a “by-product” of the meat and dairy industry (Weber et al., 2021). The scientific limitations of animal-derived materials stem from their non-human origin, potential toxicity, risk of contamination, undefined composition, and batch-to-batch variability, which have gradually, though far too slowly, come to light. In fact, many of these were *de facto* mentioned by Puck et al., in 1958 when FBS was introduced into cell culturing (Puck et al., 1958). These factors ultimately affect experimental outcomes, and they are probably part of the reported reproducibility crisis in *in vitro* cell culturing (Barosova et al., 2021). Another aspect should be the verification of the authenticity of cell lines to mitigate irreproducibility by preventing mislabeling and cross-contamination (Weiskirchen, 2026). Furthermore, the use of animal-derived materials in cell culturing during pre-clinical studies may contribute to poor translation of results and thus to the high attrition rate of new drugs (Bramwell et al., 2024). The use of non-animal methods in science offers scalable solutions that are often time-saving and economically advantageous (Hartung, 2026). However, we wish to take this further and propose that the economic advantages will be even greater with reliable, robust, reproducible, and truly animal-free methods and materials. Even though manufacturers could devise measures to address the scientific limitations of animal-derived materials, this would in turn lead to an increase in production costs and, consequently, retail prices. Nonetheless, since this would not solve the underlying ethical concerns of animal-derived materials, a change cannot be postponed any further. Hence, the call for their replacement by animal-free materials prevails, and such materials should be introduced across all research fields.

As long as animal-free methods contain animal-derived materials, they are not truly animal-free. Truly animal-free experiments can be achieved by using cell culture protocols that rely exclusively on animal-free materials. The central challenge is to reduce animal testing while humanizing cell culture systems, so they better mimic the true environment in the human body. To achieve this, long-standing “black boxes” in *in vitro* biology must be replaced with transparent, highly defined, and human-relevant culture conditions (Rosolowski et al., 2025). This Research Topic, “*Advancing in vitro cell culture practices: achieving truly animal-free experiments and scientifically reliable and reproducible methods*” was initiated and launched exactly against this background. The goal of this Research Topic is to collect, present, and disseminate studies with practical experimental setups for the use of non-animal products in different experimental settings involving cells grown in culture across a variety of conditions.

The articles show that the transition to truly animal-free cell culturing is no longer merely a concept and is becoming technically feasible across a wide range of laboratory techniques. At the same time, it is clear from the published article collection that the final goal of developing truly animal-free cell culture practices has not yet been fully achieved in all settings. It was demonstrated that some studies could achieve complete replacement of animal-derived materials, whereas in other studies, truly animal-free materials have been partially replaced. Taken together, the publications presented here have revealed progress toward the use of animal-free cell culture systems and aim to encourage and stimulate the true use of truly animal-free experimental approaches.

## Overview of the Research Topic

The Research Topic includes both review articles and original research papers demonstrating partial and complete replacement strategies. Together, they provide a broad overview of the current state of the field, highlight progress already made, and remind us of the challenges that still need to be addressed.

## Reviews

Despite the long-term use of animal-derived materials in cell culturing, the knowledge of the resulting issues has still not fully reached the broader scientific community. Therefore, the review articles in this Research Topic will help disseminate information and provide solutions.

Weber et al. elaborate comprehensively on the production, widespread use, and substitution of FBS. The review addresses FBS replacements in diverse applications such as cultivating established cell lines, stem cell culture, organoids, organ-on-a-chip systems, and bioprinting, as well as cell cryopreservation, cultivated meat production, antibody and vaccine manufacturing, and its role in toxicological research and testing.

Meng and Day examine the levels of known components of FBS and expand on recent developments in application-specific replacements, improvements in dedicated formulations, and the optimization of existing alternatives. They also underscore the establishment of databases and guidance for selecting appropriate substitutes, as well as incentive mechanisms that may further facilitate the transition away from FBS.

One viable and promising replacement for FBS is human platelet lysate (hPL), which is discussed by Immalaraju et al. in their work on hPL standardization and its comparison with FBS for human hematopoietic cell culture. The authors identify gaps in key production parameters that may influence hPL performance and recommend addressing these issues to reduce variability.

Furthermore, the review by Chaudhary and Villa-Diaz demonstrates the advancement of human pluripotent stem cell culture systems, emphasizing the progression from feeder-dependent and xenogeneic matrices toward chemically defined, xeno-free, and fully synthetic platforms. This addresses long-standing challenges in reproducibility, safety, and clinical translation, driving progress in the field toward more humanized cell and tissue culture conditions.

The comprehensive analysis by Spänle et al. of the components in human cell-based assays within the developmental neurotoxicity *in vitro* battery, provides a discussion of both the challenges and the emerging solutions associated with shifting to animal-free materials. They highlight ongoing efforts to replace animal-derived extracellular matrices, antibodies, dissociation agents, and reagents used in immunofluorescence staining.

In addition, Liefting and Bajramovic present how replacing animal-derived materials can lead to creative solutions. Because FBS is traditionally used for the production, stabilization, and solubilization of the growth factor Wnt3A, their mini-review explores alternative strategies to induce Wnt signaling in FBS-free organoid expansion media, including the use of soluble Wnt mimetics, carriers, and small-molecule inhibitors.

A novel perspective on microphysiological systems is provided by Weener et al., who describe a case study of microthrombosis in a blood-perfused vessel-on-chip model. They emphasize key elements such as defining a clear context of use, establishing relevant readouts, ensuring model robustness, and addressing inherent limitations, while underscoring that scientifically reliable models must be carefully validated.

The review by Wenzel et al. discusses the evolving landscape of recombinant antibody production systems and their applicability across various fields. The article draws attention to the emergence of new host organisms that enable the production of antibodies with characteristics that closely resemble posttranslational modifications found in the human system, without relying on animal-derived source materials.

## Replacing fetal bovine serum

Replacing FBS and other animal-derived components is often not straightforward, as clearly illustrated by the articles in this Research Topic. The authors apply diverse strategies to reduce or remove animal-derived materials, and when these approaches do not entirely succeed, they transparently report the limitations. This openness is very valuable, as it will hopefully help bring down the current technical barriers and support the development of truly animal-free cell culture systems.

Di Leonardo et al. aimed to optimize the cultivation of the Atlantic sturgeon larval cell line AOXlar7y under short-term, serum-reduced conditions. Although complete replacement of FBS was not achieved, the study presents a promising strategy for reducing serum concentration substantially by supplementing the medium with defined mouse and human growth factors and cytokines of murine origin. As fish cell lines become increasingly widely used in aquatic science, the next step should be the commercial production of recombinant fish-derived proteins to allow truly xeno-free research.

The aim of Pfeifer et al. was to culture the HepG2 hepatoblastoma cell line under fully serum-free conditions. As a first step toward this goal, they evaluated two commercially available serum-free media, although neither was entirely free of animal-derived components. Both media were shown to support the growth, morphology, and functional characteristics of HepG2 cells. Notably, because one of the media is open source, their study suggests that this information can be used to develop a truly animal-free, open-source medium for HepG2 cell culture.

Cochrane et al. compared three commercially available proprietary chemically defined media against media supplemented with human serum in a peripheral blood mononuclear cell system designed to assess how different immune cell populations respond to various treatments. Their results showed that although the chemically defined media provide more controlled cell culture conditions, they lack certain functional characteristics conferred by human serum. Consequently, further optimization is required to create truly animal-free, chemically defined media capable of fully replacing complex human-derived culture systems in immunological research.

In their brief research report, Miri et al. established human-relevant *in vitro* models consisting of bone marrow stem/stromal cells and human umbilical vein endothelial cells using animal-free materials. These included a commercially available human-cell-derived serum replacement, a plant-based blocker instead of bovine serum albumin, and hybridoma-derived recombinant antibodies produced with animal-free batch production. However, noting that hybridomas still depend on animal-derived B cells, the authors acknowledge hidden animal use in their work and the need for further development of truly animal-free research.

Malakpour-Permlid et al. combined a high-throughput scaffold-based 3D tumor model with a universal, open-access, animal-component-free medium to establish a humanized 3D *in vitro* drug screening system. They report the gradual adaptation procedure away from FBS-substituted medium for human HeLa cervical and MCF-7 breast cancer cells, as well as human cancer-associated fibroblasts, while closely monitoring cell attachment, proliferation, and morphology. Their results showed sustained growth kinetics and maintenance of population doubling times, demonstrating the feasibility of transitioning even complex multicellular models to animal-component-free conditions.

While media can be designed as universally usable formulations, Nessar et al. focused on developing a cost-efficient, open-access, and animal-component-free medium specifically for HeLa cells. A key objective of their work was to advance animal-component-free teaching by creating a graduate-level laboratory course that relied entirely on reagents free of animal-derived materials. In addition, they provided open-access protocols, allowing fully animal-free workflows to be demonstrated effectively in educational settings and helping instructors integrate such methods spanning from cell culture to molecular analysis into modern laboratory training.

## Replacing basement membrane extracts

Several studies in this Research Topic focus on the replacement of murine-derived basement membrane extracts, commonly known under the trade names Matrigel, Cultrex, or Geltrex (3Rs Centre Utrecht, 2025). For their production, mice are injected with Engelbreth-Holm-Swarm sarcoma and killed once the resulting tumor reaches almost 20% of their body weight (Berg and Kurreck, 2021).

Nitsche et al. grew the human hepatic cell line HepaRG in a microphysiological system device using several animal-free hydrogels. Cells grown in a synthetic peptide hydrogel showed promising metabolic competence under perfusion, making it a potential candidate for xenobiotic metabolism studies. However, the cell culture medium contained animal-derived materials, implying that the study is considered a partial replacement, which is a challenge that the authors address in the article.

Koivunotko et al. used a plant-derived nano-fibrillated cellulose hydrogel in an animal-free 3D *in vitro* angiogenesis model. They observed the establishment of physiologically relevant vascular networks with the occurrence of capillary-like structures. This study demonstrates the suitability of plant-based biomaterials for advancing angiogenesis modeling and for complementing established *in vitro* systems, with long-term potential to support translational biomaterial development and relevance to regenerative therapies, toxicological testing, and drug screening.

Stein and Braid presented an animal-component-free bioprocess to synthesize 3D human matrix scaffolds using mesenchymal stromal cells from placental, umbilical cord, bone marrow, and adipose tissues. Their application uses cells as a platform to produce self-assembling basement membrane components under chemically defined and xeno-free conditions. This establishes mesenchymal stromal cells as a scalable, sustainable, and cruelty-free platform for manufacturing human basement membrane matrices for bioengineering and regenerative medicine applications.

Another approach was achieved by Hayden et al., who produced a full-thickness human skin model using 3D electrospun scaffolds from biocompatible polymers in animal-component-free culture media. Testing in two independent laboratories demonstrated that these models exhibited improved lifespan and barrier properties, while also confirming protocol transferability and intra- and inter-lot reproducibility. These scaffolds can enable the development of further epithelial tissue models, such as ocular, airway, and intestinal systems.

## Discussion of the contributing articles

Taken together, the publications in this Research Topic support three broad conclusions. First, animal-derived cell culture components should no longer be viewed as “gold standards” due to scientific limitations and ethical concerns. In fact, we believe that the term “gold standard” should be avoided, as it implies a fixed and unchanging benchmark, whereas scientific knowledge and methodologies are continuously evolving.

Second, an increasing number of truly animal-free replacement alternatives are now available across different applications of cell culture, including serum supplementation, extracellular matrices, scaffolds, antibodies, and other laboratory materials. Third, we believe that the next phase of improvement will depend on standardization, transparency, and reproducibility across laboratories.

The studies in this Research Topic demonstrate progress in implementing the 3Rs (replacement, reduction, and refinement) in the life sciences, particularly by showing that *replacement* should not only involve shifting from the use of animal experiments to non-animal models. It should also ensure that these models are truly animal-free and do not rely on animal-derived components. Collectively, the contributions highlight a growing shift toward the use of truly animal-free materials in scientific research. Equally important, when animal-derived materials are still used, the authors in this collection have critically assessed the limitations of their systems and identified areas where further improvements are required. These include the continued use of proprietary media, other biomaterials, animal-derived ingredients, and incomplete replacement methods. Such shortcomings reported in several articles regarding animal-component-free conditions should not be viewed as constraints, but rather as an honest reality of the current state of the field.

## Transitional obstacles

Although we believe that the long-term goal of science should be to develop species-specific *in vitro* models that increase physiological relevance, not only for human cells but also for cells from other species, such as fish, we fully acknowledge that there are many obstacles to achieving this goal. Thus, even when researchers and laboratories wish to adopt animal-free materials, various factors can hinder progress. These include ethical perspectives that differ among nations, cultures, and individuals; academic competition; employment conditions; institutional policies; and governmental regulations. Such barriers exist at multiple levels, including the individual researcher, laboratory, institution, society, and government.

## Conclusion

In summary, the contributions in this Research Topic show clear progress toward truly animal-free *in vitro* experiments, even if the goal has not yet been fully reached in every research project. Several studies demonstrate that complete replacement is already possible in certain fields, and further studies show that some work still needs to be done to eliminate hidden animal-derived components. Nevertheless, when scientific challenges arise, solutions will evolve that complement and often revolutionize research and development, leading to new business development and work opportunities.
